# The contribution of audition and proprioception in unisensory and multisensory target reaching

**DOI:** 10.1038/s41598-025-28203-2

**Published:** 2025-12-17

**Authors:** Ivan Camponogara

**Affiliations:** College of Natural and Health Sciences, Department of Psychology, Zayed University, Abu Dhabi, UAE

**Keywords:** Auditory, Proprioceptive, Audio-Proprioceptive, Reaching, Multisensory, Sensorimotor processing, Human behaviour

## Abstract

Everyday actions often involve reaching for targets sensed by auditory and proprioceptive senses (reaching a ringing smartphone in the dark or tapping on it while holding it with the other hand). However, it is still unclear whether reaching performance toward auditory and proprioceptive targets is modality-specific and whether performance improves under multisensory compared to unisensory conditions. Here, we addressed these questions by measuring reaching performance toward auditory, proprioceptive, and combined audio-proprioceptive targets along the azimuth and depth dimensions. Accuracy was similar along the azimuth dimension, whereas precision was generally lower for auditory targets compared to proprioceptive and audio-proprioceptive targets. A second experiment investigated whether providing additional proprioceptive information while reaching for an auditory target could improve precision in subsequent auditory-only trials. A slight general improvement was observed, indicating that proprioceptive cues may help reduce spatial variability in auditory-guided actions, though not to the level seen in the multisensory condition. Overall, the results suggest that while the target modality slightly impacts movement accuracy, it has a significant impact on movement precision, with proprioceptive input playing a crucial role in enhancing precision. The concurrent availability of auditory and proprioceptive target information does not enhance precision beyond that achieved with proprioceptive information alone, whereas proprioception can modestly improve subsequent auditory-guided reaching.

## Introduction

In everyday life, humans constantly use auditory and proprioceptive information to localize objects and execute goal-directed actions. For instance, when silencing a smartphone alarm upon waking, we use auditory cues to locate and reach the smartphone in the dark and rely on proprioceptive feedback from the hand holding it or both modalities (i.e., proprioception and the ringing sound) to tap on it with the other hand and silence it. Although both sensory modalities support the same goal (i.e., successfully reaching a target), the mechanisms underlying their sensorimotor processes remain poorly understood. According to the perceptual mechanisms characterizing each sensory modality, the sensorimotor processes involved in auditory and proprioceptive target reaching may be modality-specific. Auditory localization primarily relies on interaural time and level differences, which enable relatively accurate identification of sound sources along the azimuth (i.e., horizontal) dimension^1–4^. However, localization accuracy diminishes along the depth dimension, where sound distance is often misjudged^1^. Proprioceptive localization, such as identifying the position of an unseen limb, depends on spatial encoding in joint-centered coordinates^5^. Proprioceptive judgments tend to be biased in both azimuth and depth, though exhibiting higher precision in the depth dimension than in azimuth^5–14^. Taken together, these studies suggest that actions toward auditory and proprioceptive targets may show modality-specific patterns of accuracy and precision, shaped by the distinct encoding strategies of each sensory system.

A number of studies have examined reaching movements toward either proprioceptive or auditory targets, but seldom both within the same experimental context. Consequently, several inconsistencies arise when comparing findings across studies that independently investigate reaching movements within different sensory modalities. Some studies on proprioceptive target reaching suggest more accurate and precise movements^15–19^ compared to those toward auditory targets^20–22^, across both azimuth and depth dimensions. In contrast, other studies suggest a similar accuracy or precision along either the azimuth or depth dimension^23–25^. The limited number of studies comparing reaching movements toward auditory and proprioceptive targets suggests that proprioceptive-guided actions tend to be more accurate and precise than auditory-guided actions^26,27^. Notably, in these studies, proprioceptive target reaching involved relocating a limb to a previously sensed position. Participants were first guided, either actively or passively, from a start location to a target, after which the limb was withdrawn and subsequently moved to reproduce the remembered location. This task relies not only on proprioceptive input but also on sensorimotor memory of the preceding movement^28–34^, which has been shown to significantly enhance both accuracy and precision^15^. Integrating proprioceptive feedback with sensorimotor memory likely enhances spatial estimation, potentially explaining the observed advantage of proprioceptive over auditory-guided reaching in these paradigms. Furthermore, in these studies, movement was often constrained on a rail along one movement dimension (i.e., oblique, 45 degrees), which likely reduced the natural variability inherent in skilled motor behavior^35^. Thus, discrepancies among studies, combined with the limited direct experimental comparisons between actions toward auditory and proprioceptive targets, limit our ability to conclude whether reaching movements are modality-specific.

A further open question concerns whether a multisensory advantage occurs when both sensory modalities are simultaneously available. Studies in the visuo-proprioceptive domain showed improved action performance in the multisensory condition compared to each of the unisensory conditions^36^. However, in the audio-proprioceptive domain, it remains unclear whether the concurrent availability of both auditory and proprioceptive target information enhances motor performance. Previous studies addressing multisensory-motor training on auditory localization showed that the multisensory-motor association between proprioceptive and motor signals from the reaching arm and the auditory target improves subsequent auditory localization^26,27,37–42^. This suggests that the concurrent availability of auditory and proprioceptive inputs may lead to better performance than each unisensory input alone. In contrast, studies on auditory-proprioceptive localization suggest a strong influence of arm proprioception on auditory perception when localizing a handheld speaker^43^. It is thus plausible to think that, in multisensory contexts, actions are primarily guided by proprioceptive information.

To address these questions, we conducted an experiment in which participants performed reaching movements toward targets defined by audition, proprioception, or both sensory modalities, presented at three azimuthal locations. Performance was evaluated in terms of both accuracy and precision along the azimuth and depth dimensions to assess the relative contribution of each sensory modality to action guidance and to determine whether their concurrent availability confers a performance benefit. Based on prior findings, two hypotheses can be advanced: 1) Actions toward auditory and proprioceptive targets may exhibit similar accuracy and precision along specific movement dimensions^9–13,20–22,24,25,44^. In contrast, 2) actions toward proprioceptive targets may be more accurate and precise across both dimensions^15–19^. Furthermore, if a multisensory advantage exists^26,27,37–42^, we expected that reaching in the combined audio-proprioceptive condition would exhibit higher accuracy and precision than either unisensory condition. Conversely, it may be that, in the multisensory condition, motor performance may rely on proprioceptive target information^43^, thus leading to similar action performance as in proprioceptive target reaching.

## Experiment 1

### Material and method

#### Participants

We tested twenty-five participants (right-handed, 1 male, age $21.3 \pm 1.9$ years). All self-reported normal hearing and no known history of neurological disorders. All of the participants were naïve to the purpose of the experiment. The experiment was undertaken with the understanding and informed written consent of each participant, and the experimental procedures were approved by the Institutional Review Board of Zayed University, Abu Dhabi, in compliance with the Code of Ethical Principles for Medical Research Involving Human Subjects of the World Medical Association (Declaration of Helsinki).

#### Apparatus

**Fig. 1:**
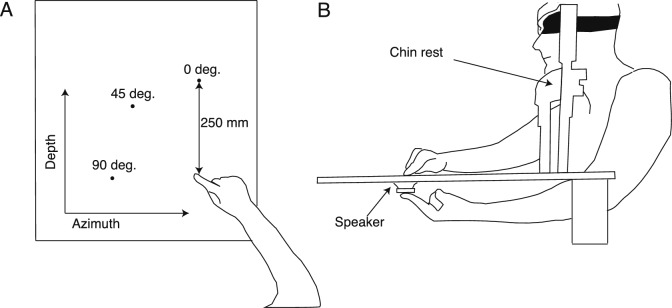
Experiment setup. (**A**) Top view: Targets were positioned at 250 mm in front (0 degrees) or laterally (45 and 90 degrees) from a start position. (**B**) Side view of the P and AP conditions: Participants placed the left index underneath the center of one of the three speakers. As soon as they heard the start sound (P) or the pink noise (AP), participants were instructed to reach the tip of their left finger (P condition) or the point where they heard the sound and felt the finger position (AP condition).

The custom-made experimental apparatus consisted of three speakers (Gikfunk, 4O 0hm 3 Watt, 40 mm diameter, 30 mm height) placed underneath a 600 $\times$ 600 mm perforated wooden board, which was supported by four 100 mm high pedestals and positioned on the experimental table. The board was uniformly perforated across its entire surface with 2 mm holes separated by 5 mm, rather than only at the speaker locations. This allowed sound transmission while preventing participants from using localized holes as tactile feedback when landing on the speaker’s position. The speakers were powered through a USB cable and connected with an external soundcard (Motu 4Pre) via a mini amplifier (PAM8403). The center of the speakers was placed 250 mm far at 0 (i.e., in front), 45, and 90 degrees on the left with respect to a 5 mm high rubber bump that acted as a starting point. The bump was placed 100 mm far from the side of the wooden board closest to the subject position (Fig. 1 A). A 5 mm high rubber bump was placed underneath the center of each speaker and was used as a reference for the left index position during the proprioceptive and audio-proprioceptive conditions. The right-hand index finger movement and the speakers’ positions were acquired at 60 Hz with a resolution of 720 X 1280 pixels using a webcam (Logitech BRIO Ultra) placed 600 mm over the wooden board with a camera tripod. The x and y pixel coordinates of the finger’s movement and speakers’ positions were extracted offline by using DeepLabCut^45^, a pose estimation toolbox system in Python. Finally, a chin rest was placed on the front side of the wooden board to avoid head movements during the experiment. The chin position was set at 25 cm height from the wooden surface, and the chair height was adjusted at the participants’ comfortable height. A pink noise of 1000 ms duration (65 db) was used as the auditory target, whereas a 100 ms duration pure tone of 500 Hz (65 dB) was used to signal the end of the trial. The same tone was used to signal the start of the trial in the proprioceptive condition. Pure tones were delivered through two external speakers (Logitech 980-000816 Z150). The camera and sound delivery were controlled via MATLAB (MathWorks), which was installed on a Dell Alienware laptop.

#### Procedure

Participants were blindfolded for the whole duration of the experiment. All the trials started with the participant’s head on the chin rest, the index digit of the right hand positioned on the start position, and the left hand on the left side of the chin rest. In an Auditory condition (A), the pink noise was delivered, and participants had to reach the point where they felt the noise coming from. In a Proprioceptive (P) and Audio-Proprioceptive (AP) condition, before each trial, participants were instructed to move the index of the left hand underneath the specified target speaker over the bump placed at the center of it. After 1 second, in the P condition, the start sound was delivered from the external speakers, whereas in AP, the pink noise was delivered from the speaker over the left finger. As soon as participants heard the sound (P) or the pink noise (AP), they had to reach the point where they thought the tip of their left index finger was, or both the tip of the finger and the pink noise were (Fig. 1 B). In all the sensory conditions, after 2 seconds from the pink noise or the start tone, the end tone was delivered, and participants had to move their right hand back to the start position and their left hand back to the side of the chin rest in P and AP conditions. Before each experiment, participants underwent a practice phase in all the conditions, consisting of 10 trials, where they got accustomed to the task. We performed 20 trials for each target, leading to 60 trials per condition (180 trials per participant). Conditions were pseudo-randomized, with A always being the first. Then, half of the participants underwent the P and AP order, and the other half the AP and P order. This randomization was chosen to prevent trials in A condition to be affected by any multisensory learning effect if AP and P conditions were performed at first^26,27,37,38,40–42^.

#### Data analysis

Kinematic data were analyzed in R^46^. The x and y coordinates of the index and speakers’ position in pixels were smoothed and differentiated with a third-order Savitzky-Golay filter with a window size of 21 points. These filtered data were then used to compute velocities and accelerations of the index finger in two-dimensional space. Movement onset was defined as the moment of the lowest, non-repeating index finger acceleration value prior to the continuously increasing index finger acceleration values, while the end of the movement was defined by applying the same algorithm but starting from the end of the recorded values^14^. Subject 7 voluntarily withdrew from the experiment when in the P condition, performing 32 trials in total instead of 60. From the 4348 trials, we discarded from further analysis the trials in which the camera (48 trials) or the algorithm (137 trials) did not capture the end of the movement. The exclusion of these trials (185 trials, 4.2% of all trials) left us with 4163 trials for the final analysis. For each trial, we calculated the endpoint positions along the azimuth and depth dimensions (i.e, the x and y coordinates).

We focused our analyses on the x and y coordinates of the endpoint error, defined as the distance between the center of the target and the index’s landing point. The x and y endpoint error coordinates were analyzed using the Bayesian multivariate linear mixed-effects distributional regression model, estimated using the brms package^47^, which implements Bayesian multilevel models in R using the probabilistic programming language Stan^48^. The distributional regression model allowed us to estimate the posterior distribution of both the accuracy and the SD, taking into account both within and between-subject variability^14^. The model included as predictors (i.e., fixed effects) the categorical variable Condition (A, P, and AP) and the continuous variable Direction (0, 45, and 90 degrees). Thus, the model estimated the average accuracy in depth and azimuth ($\beta _{Condition}$), its change as a function of the target direction ($\beta _{Direction}$, slope), the average SD in depth and azimuth $\sigma _{Condition}$, and the SD change in azimuth and depth as a function of the target direction ($\sigma _{Direction}$, slope). The model included the independent random (group-level) effects for subjects.

The model was fitted considering weakly informative prior distributions for each parameter to provide information about their plausible scale. We used Gaussian priors for the Condition fixed-effect predictor ($\beta _{Condition}$: mean = 0 and sd = 80, $\beta _{Direction}$: mean = 0 and sd = 5, $\sigma _{Condition}$: mean = 2.5 and sd = 2,$\sigma _{Direction}$: mean = 0 and sd = 2), whereas for the group-level standard deviation parameters we used zero-centered Student *t*-distribution priors, with scale values defined by the “get_prior” function. (azimuth = *df* = 3, scale = 32.9, depth = *df* = 3, scale = 34.5). Finally, we set a prior over the correlation and residual correlation matrix that assumes that smaller correlations are slightly more likely than larger ones (LKJ prior set to 2).

We ran four Markov chains simultaneously, each for 4,000 iterations (1,000 warm-up samples to tune the MCMC sampler) with the delta parameter set to 0.99 for a total of 12,000 post-warm-up samples. Chain convergence was assessed using the $\hat{R}$ statistic (all values equal to 1) and visual inspection of the chain traces. Additionally, the predictive precision of the fitted models was estimated with leave-one-out cross-validation by using the Pareto Smoothed Importance Sampling (PSIS). We found 1 pareto k value higher than 0.7, and re-fit the model using the reloo function in brms^47^. After the model re-fit, all the pareto k values were lower than 0.7.

The posterior distributions we have obtained represent the probabilities of the parameters conditional on the priors, model, and data, and they represent our belief that the “true” parameter lies within some interval with a given probability. We summarize these posterior distributions by computing the medians and the 95% Highest Density Intervals (HDI). The 95% HDI specifies the interval that includes, with a 95% probability, the true value of a specific parameter. To evaluate the differences between the two compared conditions, we have simply subtracted the posterior distributions of $\beta _{Condition}$, $\beta _{Direction}$, $\sigma _{Condition}$, and $\sigma _{Direction}$ between specific conditions. The resulting distributions are denoted as the credible difference distributions and are again summarized by computing the medians and the 95% HDIs.

For statistical inferences about the $\beta _{Condition}$, $\beta _{Direction}$, $\sigma _{Condition}$ and $\sigma _{Direction}$ we assessed the overlap of the 95% HDI with zero. A 95% HDI that does not span zero indicates that the accuracy ($\beta _{Condition}$) and SD ($\sigma _{Condition}$) in depth and azimuth were credibly different than zero in that specific condition. Additionally, a 95% HDI that does not span the zero for the $\beta _{Direction}$ and $\sigma _{Direction}$ indicates that both the accuracy and SD, respectively, are modulated according to the target direction. For statistical inferences about the differences between conditions, we applied an analogous approach. A 95% HDI of the credible difference distribution that does not span zero is taken as evidence that the accuracy ($\beta _{Condition}$) or SD ($\sigma _{Condition}$) in the two conditions differ from each other. The same applied to the predictor Direction. To assess the strength of the evidence, we computed the Bayes factor for each comparison. The reported Bayes factor values ($\hbox {BF}_{\text {10}}$) that are higher than 3 provide evidence in support of a difference between conditions, whereas values below $\frac{1}{3}$ provide evidence in support of an absence of a difference between conditions^49^.

**Fig. 2:**
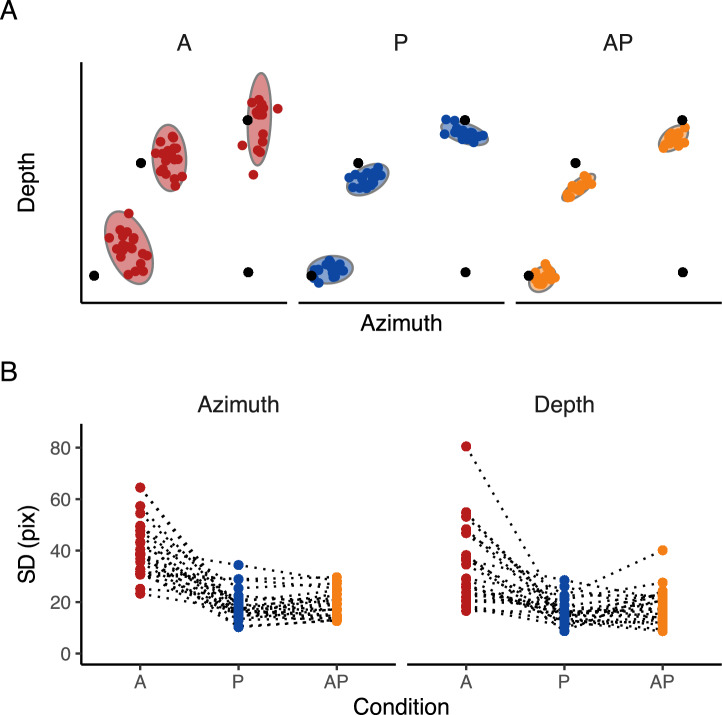
Raw data. (**A**) Final landing points (colored dots) and their 95% confidence ellipses standard deviation of a representative participant for experiment 1. The bottom right black dot represents the positions of the starting point, whereas the other three black dots represent the center of the three targets. (**B**) Raw SD. Raw SD of each participant in A, P, and AP conditions in the azimuth and depth dimensions. Dotted lines connect each participant’s result in each condition.

### Results and discussion

**Fig. 3:**
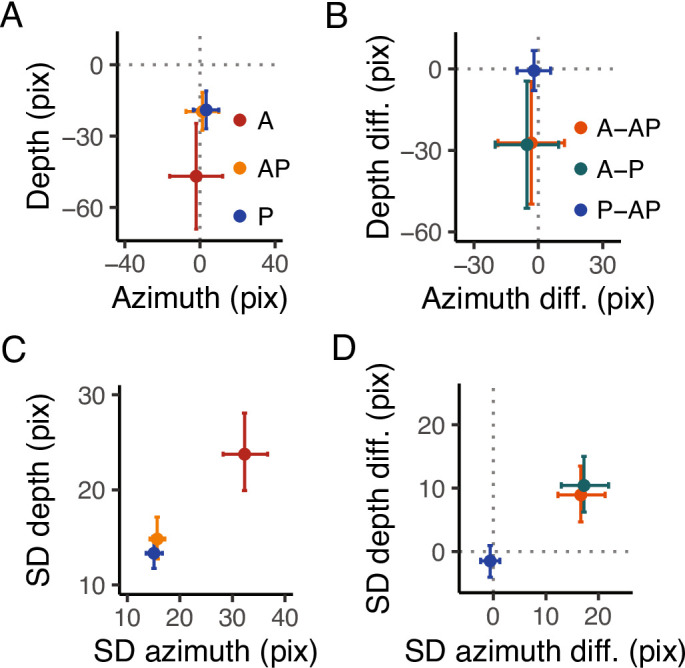
Experimental results. (**A**) Estimates of the accuracy in the azimuth and depth. The dots represent the mean, and the error bars denote the 95% HDI of the estimates. The intersection of the grey dotted lines represents the center of the targets. In A, positive values in the depth dimension indicate an overshoot, whereas positive values in the azimuth indicate that the index finger landed on the right side of the target. (**B**) Estimates of the differences in accuracy in the azimuth and depth between conditions. The dots represent the mean, and the error bars denote the 95% HDI of the estimates. The grey dotted lines represents the points of equal error in the depth and azimuth dimensions. (**C**) Estimates of the SD in the azimuth and depth. The dots represent the mean, and the error bars denote the 95% HDI of the estimates. (**D**) Estimates of the differences in SD in the azimuth and depth between conditions. The dots represent the mean, and the error bars denote the 95% HDI of the estimates. The grey dotted lines represents the points of equal variability in the depth and azimuth dimensions.

In general, we found an overall target undershoot across conditions (Figs. 2A, 3A). The accuracy was credibly similar between conditions (Fig. 3B), with a minimal difference between A-AP (depth = −27 pixels, HDI = −49.7, −4.67 pixels; azimuth = −3 pixels, HDI = −18.8, 12.2 pixels) and A - P (depth = −27 pixels, HDI = −51.3, −4.47 pixels; azimuth = −5 pixels, HDI = −20.2, 9.41 pixels) along the depth dimension. However, all $\hbox {BF}_{\text {10}}$ were < 3, indicating anecdotal evidence toward the alternative hypothesis. The slope analysis revealed a credible similar accuracy across target directions for P and AP (i.e., flat slope), whereas there was a slight increase in the accuracy along the depth dimension for the A condition, with, however, an anecdotal evidence toward the alternative hypothesis ($\hbox {BF}_{\text {10}}$ = 0.3). No credible difference for the slope was found between conditions.

The SD (Figs. 2B, 3C and D), instead, was credibly lower with strong evidence toward the alternative hypothesis in A compared to AP in both the depth ($\hbox {BF}_{\text {10}}$ >100) and azimuth dimensions ($\hbox {BF}_{\text {10}}$ >100). The same held for the comparisons between the A and P conditions in both depth and azimuth ($\hbox {BF}_{\text {10}}$ >100). The comparisons between P and AP revealed a credibly similar SD (all $\hbox {BF}_{\text {10}}$ < 1). The slope showed a minimal effect of target direction on SD, showing a slight decrease of SD in A and a slight increase in P as the target is laterally displaced (i.e., 90 degrees) along the depth dimension, whereas along the azimuth in P and AP. However, such changes were irrelevant (all $\hbox {BF}_{\text {10}}$ < 1). The analysis between conditions showed a minimal difference in slope between P and AP and between A and P in the depth (P-AP: 0.04 pixels, HDI = 0.004, 0.08 pixels, A-P: −0.09 pixels, HDI = −0.15, 0.03 pixels) dimension, and between A and AP, and A and P in the azimuth dimension (A-AP: 0.15 pixels, HDI = 0.004, 0.3 pixels, A-P: 0.15 pixels, HDI = 0.01, 0.3 pixels). However, all $\hbox {BF}_{\text {10}}$ were < 1, indicating anecdotal evidence toward the alternative hypothesis.

#### Conclusion experiment 1

Taken together, results showed a credibly similar accuracy in A, P, and AP, confirming a similar accuracy across conditions^5,20–22,50–52^ in the azimuth dimension, and a slightly higher bias for the depth dimensions in auditory target reaching^1,53^. Actions toward proprioceptive and audio-proprioceptive targets were more precise than those toward auditory targets, hinting at a higher reliance on proprioception for action consistency when reaching multisensory targets. Such a process raises the question of the effectiveness of additional proprioceptive target information in ameliorating subsequent reaching performance toward an auditory target. Several studies in the literature reported a better auditory target localization following sensorimotor (proprioceptive and motor) training, where participants actively or passively reached an auditory target^26,27,37,38,40–42,54,55^. Such studies suggested that the improved performance after the training was due to a cross-modal effect, where sensorimotor inputs from the reaching arm are used to map the auditory target location. In other words, the improved auditory localization performance after the auditory target reaching may be due to the combination of proprioceptive and motor inputs signaling the final extension of the reaching arm with the auditory target location^26,37^. It is thus still unclear whether adding proprioceptive target information may ameliorate a subsequent action performance toward an auditory target. To investigate the effect of additional proprioceptive information on auditory target reaching, we performed a second experiment where we measured the auditory target reaching accuracy and precision before (Pre) and after (Post) reaching an audio-proprioceptive target. If participants use proprioception to map the auditory target location, results will show a better action performance (higher accuracy and precision) in the Post condition compared to the Pre condition. In contrast, a lack of action improvement would prove that additional proprioceptive target information is insufficient to map an auditory target location.

## Experiment 2

### Material and method

#### Participants

We tested twenty-five participants (right-handed, 1 male, age $25.1 \pm 8.9$ years). All had normal or corrected-to-normal vision and no known history of neurological disorders. All of the participants were naïve to the purpose of the experiment. The experiment was undertaken with the understanding and informed written consent of each participant, and the experimental procedures were approved by the Institutional Review Board of Zayed University, Abu Dhabi, in compliance with the Code of Ethical Principles for Medical Research Involving Human Subjects of the World Medical Association (Declaration of Helsinki).

#### Apparatus and procedure

The apparatus and procedures were the same as Experiment 1. Participants performed an A condition, named Pre, an AP condition, and a second A condition, named Post. We run 20 trials for each target, leading to 60 trials per condition (180 trials per participant).

#### Data analysis

The raw data processing and statistical analysis were the same as in Experiment 1. From the 4500 trials, we discarded from further analysis the trials in which the end of the movement was not captured correctly by the camera (213 trials in total) or the algorithm (73 trials in total). The exclusion of these trials (286 trials, 6.3% of all trials) left us with 4214 trials for the final analysis. For each trial, we calculated the endpoint positions along the azimuth and depth dimensions. As in Experiment 1, we focused our analyses on the endpoint error. The $\hat{R}$ and visual inspection of the chain traces confirmed successful chains convergence. All pareto k values were lower than 0.7.

A further analysis was performed to define whether a learning effect occurred throughout each condition. For each participant, we split the 60 trials into 3 blocks of 20 trials each. We then analyzed the endpoint error using the Bayesian multivariate linear mixed-effects distributional regression model, with the categorical variable Condition (Pre, AP, Post) in combination with the centered continuous variable Block (1,2,3). The model included the independent random (group-level) effects for subjects. The model was fitted considering weakly informative prior distributions for each parameter to provide information about their plausible scale. We used Gaussian priors for the Condition and Block fixed-effect predictors ($\beta _{Condition}$: mean = 0 and sd = 80, $\sigma _{Condition}$: mean = 2.5 and sd = 2, $\beta _{Block}$: mean = 0 and sd = 5, $\sigma _{Block}$: mean = 0 and sd = 2), whereas for the group-level standard deviation parameters we used zero-centered Student *t*-distribution priors, with scale values defined by the “get_prior” function. (azimuth = *df* = 3, scale = 43.8, depth = *df* = 3, scale = 41). Finally, we set a prior over the correlation and residual correlation matrix that assumes that smaller correlations are slightly more likely than larger ones (LKJ prior set to 2). The $\hat{R}$ and visual inspection of the chains traces confirmed successful convergence. We found 1 pareto k value higher than 0.7, and re-fit the model using the reloo function in brms^47^. After the model re-fit, all the pareto k values were lower than 0.7. We focused our analysis on the $\sigma _{Condition}$ parameter, which corresponds to the within-participant SD in azimuth and depth dimensions in each Condition, and the estimates of the Block parameter $\sigma _{Block}$, which corresponds to the change of within-participant SD along the azimuth and depth dimension as a function of the Block (i.e., the slope). The reason for focusing on the model’s $\sigma$ component is rooted in the findings of Experiment 1, where it was observed that providing proprioceptive target information resulted in a significant reduction in the SD.

**Fig. 4:**
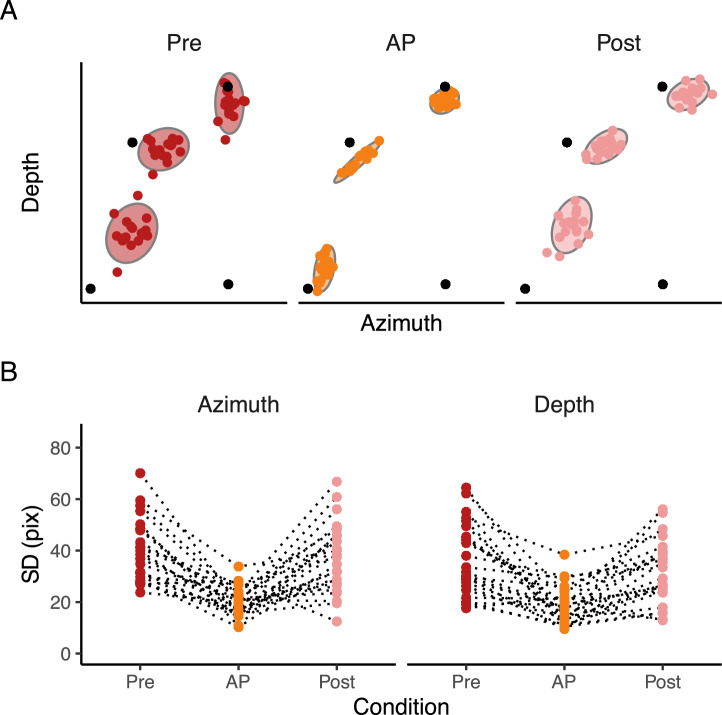
Raw data. (**A**) Final landing points (colored dots) and their 95% confidence ellipses standard deviation of a representative participant for experiment 2. The bottom right black dot represents the positions of the starting point, whereas the other three black dots represent the center of the three targets. (**B**) Raw SD. Raw SD of each participant in Pre, AP, and Post conditions in the azimuth and depth dimensions. Dotted lines connect each participant’s result in each condition.

### Results and discussion

#### Accuracy and SD

**Fig. 5:**
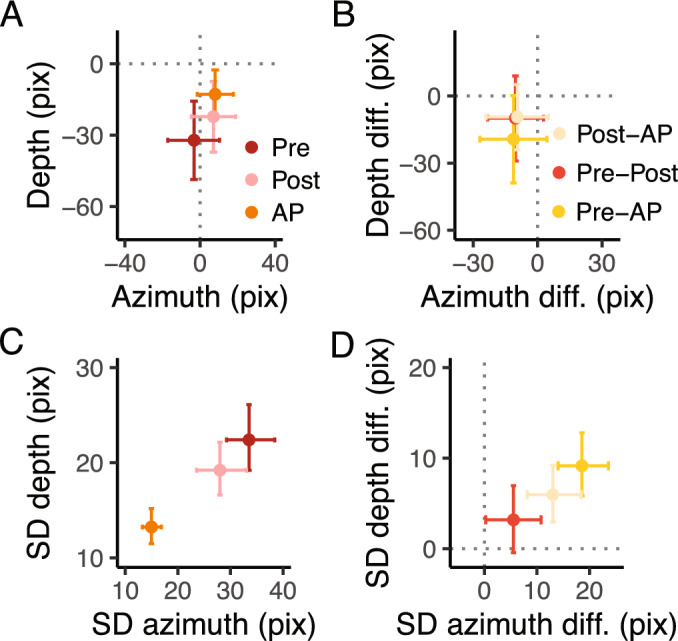
Experimental results. (**A**) Estimates of the accuracy in the azimuth and depth. The dots represent the mean, and the error bars denote the 95% HDI of the estimates. The intersection of the grey dotted lines represents the center of the targets. In A, positive values in the depth dimension indicate an overshoot, whereas positive values in the azimuth indicate that the index finger landed on the right side of the target. (**B**) Estimates of the differences in accuracy in the azimuth and depth between conditions. The dots represent the mean, and the error bars denote the 95% HDI of the estimates. The grey dotted lines represents the points of equal error in the depth and azimuth dimensions. (**C**) Estimates of the SD in the azimuth and depth. The dots represent the mean, and the error bars denote the 95% HDI of the estimates. (**D**) Estimates of the differences in SD in the azimuth and depth between conditions. The dots represent the mean, and the error bars denote the 95% HDI of the estimates. The grey dotted lines represents the points of equal variability in the depth and azimuth dimensions.

In general, we found an overall target undershoot across conditions (Figs. 4A, 5A). The accuracy was credibly similar between conditions (all $\hbox {BF}_{\text {10}}$ < 3, Fig. 5 B) and did not vary according to the target directions (all $\hbox {BF}_{\text {10}}$ < 2). As for experiment 1, there was a tendency of a higher target undershooting in the Pre compared to the AP condition along the depth dimension (Pre - AP = −19.3 pixels, HDI = −38.9, 0.1 pixels), but it was not credibly different. Even though the slope in the Pre condition was credibly higher than in the AP condition (difference = 0.3, HDI = 0.1, 0.6), we found anecdotal evidence toward the alternative hypothesis ($\hbox {BF}_{\text {10}}$ = 0.66).

The SD instead (Figs. 4B, 5C and D), was credibly lower with a strong evidence toward the alternative hypothesis ($\hbox {BF}_{\text {10}}$ >100) in Pre compared to AP (depth = 9.2, HDI = 5.84, 12.8 pixels; azimuth = 18.6, HDI = 14.0, 23.6 pixels) and in Post compared to AP (depth = 5.9, HDI = 2.9, 9.2 pixels; azimuth = 13.0, HDI = 8.1, 18.4 pixels). The SD between Pre and Post was credibly similar along the depth dimension (depth = 3.19, HDI = −0.4, 6.9 pixels), but not along the azimuth dimension (azimuth = 5.53., HDI = 0.2, 10.8). However, the BF showed very weak evidence toward the alternative hypothesis for all the dimensions ($\hbox {BF}_{\text {10}}$ depth = 0.2, $\hbox {BF}_{\text {10}}$ azimuth = 0.9). The slope for the SD was credibly lower than 0 in the depth dimension for the Pre (slope = −0.08, HDI = −0.1, −0.02 pixels) and Post conditions (slope = −0.07, HDI = −0.1,−0.02 pixels), with, however, anecdotal evidence toward the alternative hypothesis ($\hbox {BF}_{\text {10}}$ < 3). The comparison between conditions revealed a credibly lower slope in the depth dimension in the Pre compared to AP (slope diff. = −0.08, HDI = −0.1, −0.03 pixels) and in the Post compared to AP (slope diff. = −0.07, HDI = −0.1,−0.01 pixels). The Bayes factor revealed, however, anecdotal evidence toward the alternative hypothesis ($\hbox {BF}_{\text {10}}$ < 3).

In sum, results showed that reaching an audio-proprioceptive target has a minimal impact on improving action performance toward auditory targets. Moreover, even though the SD in the Post was slightly lower than in the Pre condition, it did not reach the same level as in the AP condition.

#### Within conditions learning effect

**Fig. 6:**
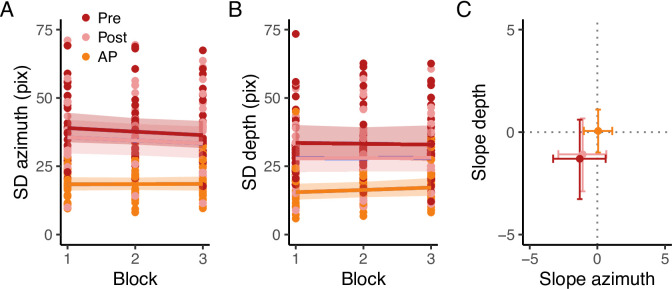
Learning effect results. Raw endpoint standard deviation in the azimuth (**A**) and depth (**B**). The dots represent the raw single participant standard deviation, whereas solid lines and shaded regions represent the estimated standard deviation and the 95% HDI of the posterior distributions of the Bayesian regression model. (**C**) Estimated slopes of the endpoint standard deviation as a function of the trial block for each condition. Points represent the mean and the error bars, the 95% HDI of the posterior distributions.

The analysis revealed a lack of learning effect (i.e., change in movement precision) across blocks (Fig. 6A and B). The slope was not credibly different than 0 in each condition, revealing no changes in the SD as a function of the trial block (Fig. 6 C). Additionally, comparison between conditions revealed a credibly similar slope across conditions (all HDIs crossing 0). This further confirms that additional proprioceptive target information is insufficient to improve action performance throughout the task. Moreover, it confirms that in the AP condition, participants mainly relied on proprioception to perform the movement.

## Discussion

In this study, we investigated how reaching movements toward auditory and proprioceptive targets compare. To this aim, we asked participants to perform a reaching task toward an auditory, proprioceptive, or audio-proprioceptive target and measured the accuracy and precision (SD) in azimuth and depth dimensions. We found that actions toward proprioceptive and auditory targets show similarities in accuracy along the azimuth dimension, with slightly better accuracy for proprioception along the depth dimension. However, actions toward proprioceptive targets were more precise (lower SD) than those toward auditory targets in both azimuth and depth dimensions. Multisensory target reaching, instead, resembles proprioceptive target reaching for both accuracy and precision, confirming a high reliance on proprioception when reaching an audio-proprioceptive target^43^.

Taken together, our results contribute to clarifying findings from studies that separately investigated proprioceptive and auditory target reaching. Specifically, results align with previous studies suggesting modality-specific patterns of accuracy and precision along the azimuth and depth dimensions^14,16,19–25^. The similar accuracy in the azimuth but a lower precision across both dimensions in auditory target reaching may be due to the inherently lower spatial resolution of binaural auditory cues compared with the joint-centered proprioceptive signals. In this regard, evidence from separate studies on auditory^1–4,56,57^ and proprioceptive localization^5,8–13,26,27,51^, also suggests modality-specific accuracy and precision, with audition being generally less accurate in depth and less precise across both spatial dimensions. This perceptual pattern is consistent with the performance differences observed in the present study, suggesting that the observed differences in reaching accuracy and precision could partly reflect inherent modality-specific characteristics.

**Fig. 7:**
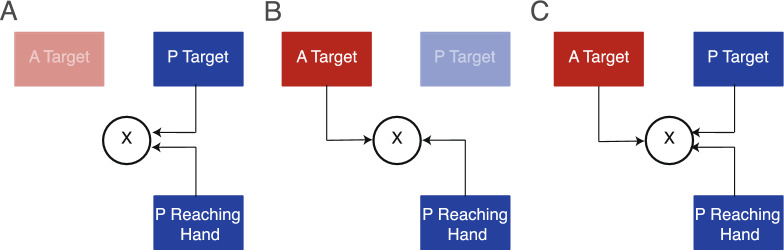
Schematic representation of the models of sensorimotor processes in unisensory and multisensory conditions. (**A**) Combination of target and reaching hand information in the proprioceptive condition. (**B**) Combination of target and reaching hand information in the auditory condition. (**C**) Combination of auditory and proprioceptive target and proprioceptive reaching hand information in the audio-proprioceptive condition.

Considering our task (i.e., reaching a target sensed through different modalities), differences between modalities could also be due to the intra- and cross-modal comparisons involved in defining the *target* and the *reaching hand *positions. In this regard, such comparisons may resemble those outlined in the Multimodal Theory of Sensory Integration for vision and haptics^58^. In the proprioceptive condition, both the *target* and the *reaching hand* positions were processed within the same sensory modality, allowing for a direct intra-modal comparison (Fig. 7A). In contrast, in the auditory condition, the comparison involved different sensory modalities: the *target* location was specified via auditory input, while the position of the *reaching hand* remained available through proprioception (Fig. 7B). This cross-modal comparison may have introduced additional variability in performance, leading to less consistent actions in the auditory condition compared to the proprioceptive one. In the audio-proprioceptive condition, proprioceptive signals conveyed information about both the *target* and the *reaching hand*, while auditory signals provided additional, redundant information about the *target* location. This involved an asymmetric distribution of sensory information, with proprioception contributing to both *target* and *reaching hand* localization, and audition contributing solely to the *target* (Fig. 7C). Such asymmetry may have led to a predominant reliance on proprioceptive information to reduce action variability.

The potential benefit of proprioceptive information on auditory target reaching was investigated in a second experiment. Here, participants performed an auditory target reaching task both before and after an audio-proprioceptive target reaching session. While the effect was not strong, results revealed a slight reduction in variability along both movement dimensions. This modest improvement contrasts with previous studies reporting stronger effects of sensorimotor training on auditory localization. Several methodological differences between the current study and previous research may account for this discrepancy. Notably, many previous studies assessed participants’ auditory localization abilities under conditions of auditory disruption, either by including individuals with hearing impairments^40,41,59^, simulating monaural hearing loss in normally hearing individuals^37,38,42,60^, or manipulating the spectral content of auditory stimuli^61–63^. In such conditions, a training session consisting of reaching the auditory target has been shown to significantly enhance a subsequent auditory localization performance^42,60,61,63^. In contrast, studies involving participants with intact hearing abilities typically report minimal or no improvements following auditory target reaching^27,64^. This suggests that training-related gains may emerge in disrupted auditory conditions when auditory reliability is compromised. The lack of substantial effects in the present study may reflect the relatively high baseline reliability of auditory inputs in our intact-hearing participants. Such effects may be more likely to emerge under conditions of impaired or degraded auditory perception.

An additional important consideration potentially explaining the small benefits of additional proprioceptive target information in our experiment relates to the use of terminal feedback in prior studies, which often involved visual^65–67^, audiovisual^37,38,40–42^, or proprioceptive and tactile cues^54,55^ to inform participants of their performance throughout the training phase. For example, in studies employing virtual reality environments, participants were presented with an array of virtual speakers. During the pre- and post-training assessments, a sound was played, and participants were asked to verbally identify the corresponding speaker or to point their head toward the speaker emitting the sound. During the training phase, participants performed a reaching movement toward the sound-emitting speaker, which continued to play until the target was successfully reached, at which point the speaker turned green and the sound ceased^37,38,40–42^. Similarly, in studies involving physical speakers, blindfolded participants were instructed to reach and touch the speaker emitting the sound^26,27,54,55^. In this case, the tactile information participants experienced at the end of the movement acted as feedback about the final speaker position. It is well established that terminal visual^68–70^ and proprioceptive feedback^71^ can drive sensorimotor adaptation^72^. The lack of any externally delivered terminal feedback in our study highlighted the specific role of additional proprioceptive target information in subsequent auditory reaching performance.

Taken together, our results show that action performance toward auditory and proprioceptive targets demonstrates similar accuracy along the azimuth dimension, with proprioception showing slightly greater accuracy along the depth dimension. However, actions differ in precision, with those directed toward proprioceptive targets being more precise than those directed toward auditory targets. In multisensory target reaching, actions heavily rely on proprioceptive target information to reduce action uncertainty. When testing the effect of additional proprioceptive inputs on a subsequent auditory target reaching performance, the slight improvement in the auditory target reaching suggests that proprioceptive inputs could contribute to reducing the variability in the auditory target location, but such an effect is not as strong as when auditory information is disrupted^37,38,41,41,42,59–63^.

### Limitations

There are several limitations to consider in the performed experiments, which are related to the use of a perforated board and the number of targets used. Even though perforated, the use of a wooden board may have attenuated the high-frequency spectrum, potentially reducing auditory reliability. Nevertheless, studies showed that precision in sound localization in the horizontal dimension decreased for limited-band high-frequency noises (3–10 kHz) compared to those within the low-frequency band (0.1–1 kHz)^56^, thus suggesting a preserved precision in the auditory condition. The higher reliance on proprioception in experiment 1 and the slight improvement seen in experiment 2 could also be attributed to the limited number of target locations (three). It is possible that, in each trial, participants relied on the memory of the left-hand position to map the targets locations, leveraging this mapping to either enhance their performance in P and AP (experiment 1) or in the post-training session (experiment 2). An alternative explanation for the improvement in experiment 2 could involve the attenuated sound frequencies produced by the perforated wooden board, which may have reduced the reliability of auditory cues and enhanced reliance on proprioceptive cues. Even though studies showed that sound localization variability is lower (i.e., higher precision) for low compared to high frequencies sounds^56^, a potential reduction in auditory reliability, combined with a limited number of auditory targets, may have led participants to increasingly depend on the previously sensed proprioceptive target positions to reach the auditory target in the post-training session.

## Data Availability

All data and data analysis scripts are available at the following link: https://osf.io/cu2q3/.
